# Supplementary figures and images for: Pancreatic Ductal Adenocarcinoma (PDAC) circulating tumor cells influence myeloid cell differentiation to support their survival and immunoresistance in portal vein circulation
Source: PLoS One. 2022 Mar 22;17(3):e0265725. doi: 10.1371/journal.pone.0265725 (PMC8939813; doi:10.1371/journal.pone.0265725)

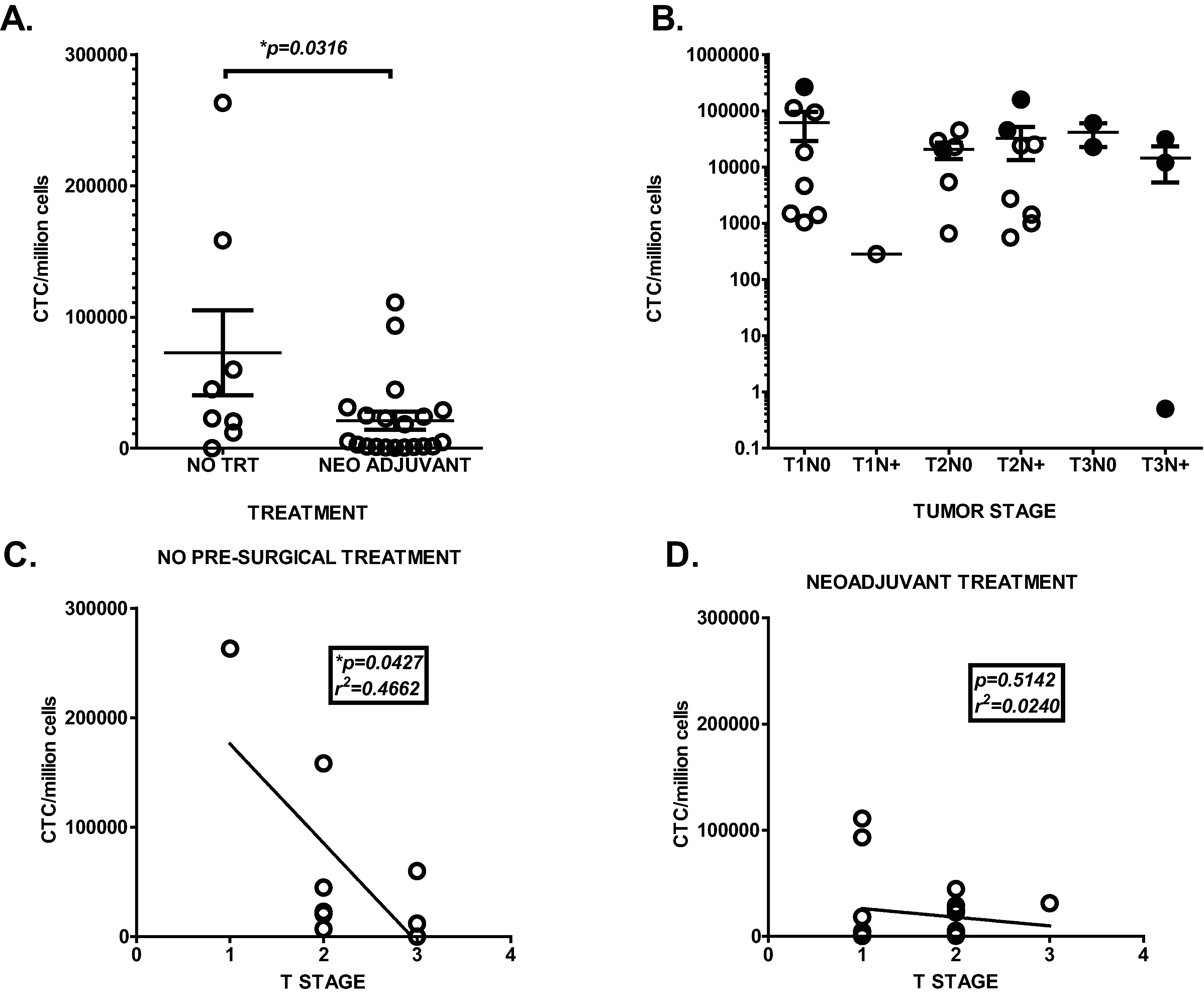

Supplement: S1 Fig — A) CTC were aseptically isolated from freshly ficoll gradient separated, uncultured portal blood mononuclear cells (PortalBMC) using high-speed FACS. CD44+CD147+EPCAM+/CK+CD45- CTC numbers were compared between 20 PDAC patients that received pre-operative treatment (Neo adjuvant chemotherapy ± radiation) and 8 patients who had no pre-operative therapy. As previously reported [7, 11], PDAC patients receiving neo adjuvant chemotherapy with or without radiation had lower portal blood CTC counts than untreated patients (p = 0.0361; with noted variability in sample population distribution). Sample values are shown by open circles, horizontal bars indicate mean of values and error bars depict standard error of the mean. B) Comparison of PDAC patient portal CTC numbers in uncultured PortalBMC sub-grouped by tumor stages (T1-3) with (N+) or without (N0) lymph node involvement. TN staging was derived from the post-operative resected tumor tissue pathological review for PDAC patients verified after analysis of portal blood samples from the same patients. PDAC Patients receiving pre-operative treatments are depicted by white circles. PDAC patients without neoadjuvant treatment are represented by black dots. Horizontal bar indicates mean of values and error bars depict standard error of the mean. No statistical difference in portal blood CTC counts was seen between tumor stage with or without lymph node involvement. C) A correlation analysis of the 8 PDAC patients without neoadjuvant therapy showed a weakly linear but statistically significant negative relationship between portal blood CTC numbers and tumor (T) staging, with more CTC detected in lower stage tumors than in higher stage tumors (p = 0.0471; r2 = 0.4662). D) Correlation analysis showed no relationship between portal blood CTC numbers and tumor (T) staging in 20 PDAC patients receiving neo adjuvant treatment. Two PDAC patients (one T1N0, one T3N0) in the study did not have pre-culture FACS isolation of CTC performed [file pone.0265725.s001.tif]

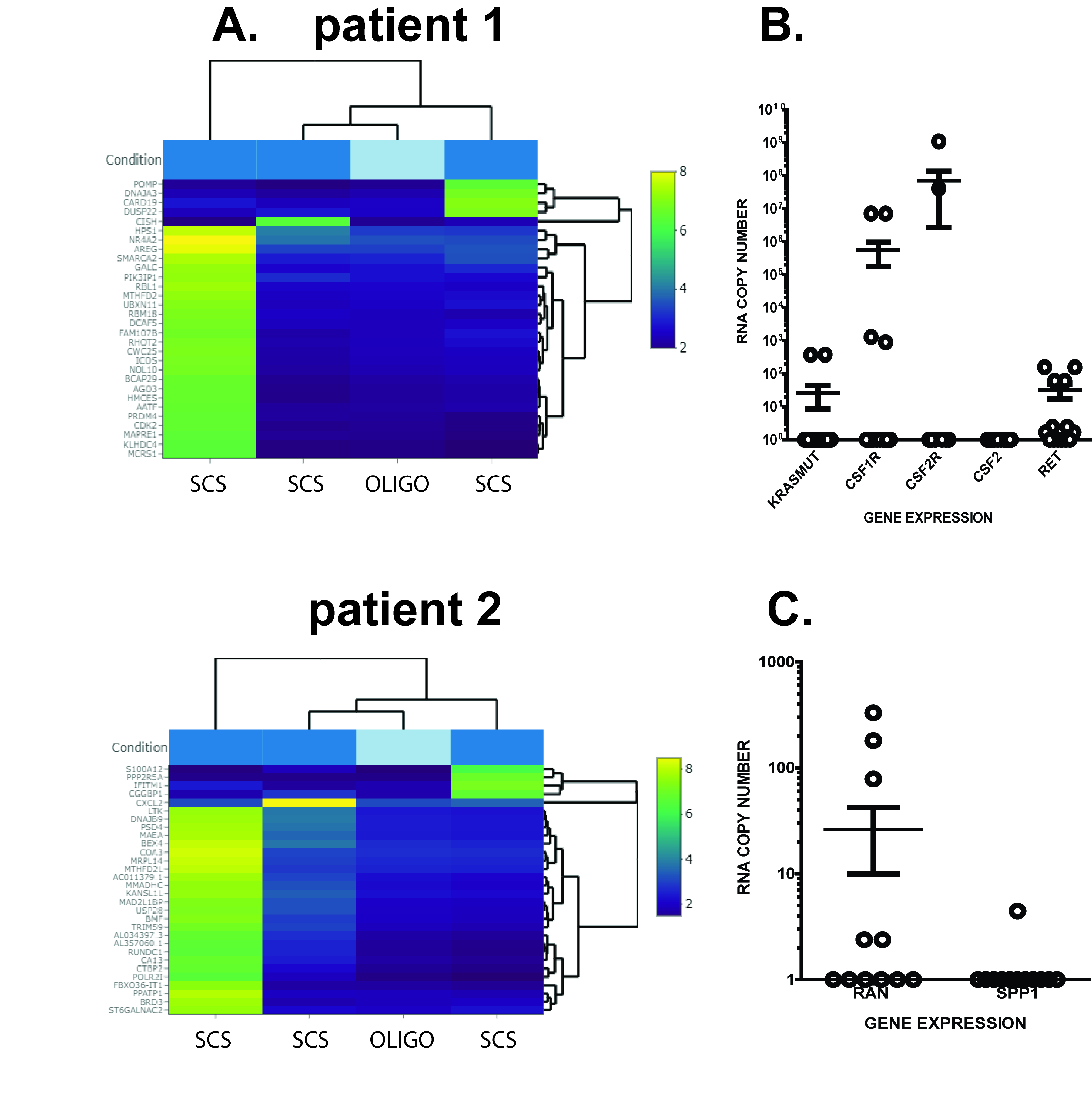

Supplement: S2 Fig — A) FACS isolated single clones (SCS) of CTC from 2 PDAC patients’ uncultured PortalBMC were collected directly into RNALater and shipped frozen to GeneWiz for RNA extraction and analysis using RNA Microsequencing (RNA SEQ, GeneWiz, NJ,USA). Analysis of the top 100 genes expressed by individual clonal CTC (SCS) and oligoclonal CTC FACS isolations (OLIGO) for each patient were compared. A) Heatmaps depict the top 30 genes found by GeneWiz analyses for 3 SCS clones and 1 sample of oligo CTC isolates for the 2 patients as examples of variability among clones of each patient. Further bioinformatic analyses of the expression patterns within each patients’ clonal variants revealed some clones with elevated expression of myeloid cell growth factor signaling genes and increased expression of the oncogene RAN and its regulatory target gene SPP1, which encodes for the tumor motility promoting protein osteopontin [S1], among the differentially expressed genes in PDAC clones. For confirmatory analyses of these candidate genes’ expression, FACS-isolated single clones of CTC from 12 patients with PDAC were individually extracted and yielded limited RNA samples for quantitative RT-PCR analysis. GAPDH RNA expression in clonal cell isolates (data not shown) was used for RNA copy number calculations of KRASmut, oncogene RET1 [S2], CSF2, CSF2R, and CSF1R expression per clone. B) Graph depicts results from analysis of CTC SCS from 12 PDAC patients using 3 CTC SCS clones per patient, confirming high expression of KRASmut, RET1, and CSF1R RNA with variability seen within each patient’s clonal set. Two CTC clones from a single patient showed high expression of CSF2R as well. C) Quantitative RT-PCR analysis of RNA from SCS CTC clones of 5 individual PDAC patient portal blood samples showed high variability of RAN oncogene expression while its substrate target gene, SPP1, remained relatively stable in its expression across clones. Sample values are shown by open circles, horizontal bar indic [file pone.0265725.s002.tif]

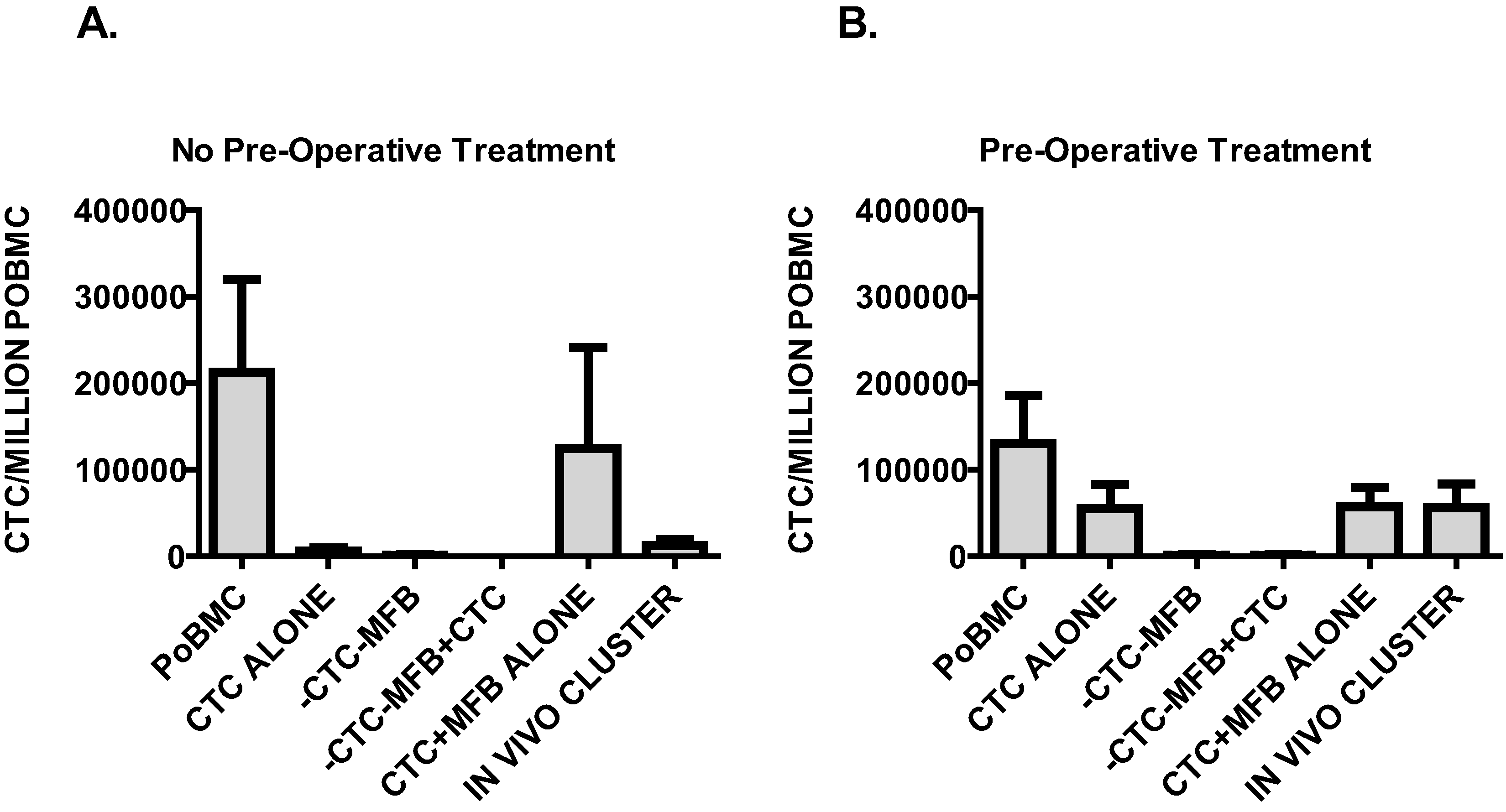

Supplement: S3 Fig — Data depicted are derived from Fig 1 panel E. Among the 16 PDAC patient samples depicted, 13 patients received neoadjuvant treatment with chemotherapy with or without radiation therapy prior to resection. The remaining 3 patients that did not undergo neoadjuvant therapy had higher CTC counts overall after ex vivo culture, but otherwise changes in CTC proliferation in the defined isolated cell ex vivo cultures were not significantly different from that seen in those of patients receiving neoadjuvant treatment. Bars indicate mean of values and error bars depict standard error of the mean. (TIF) [file pone.0265725.s003.tif]

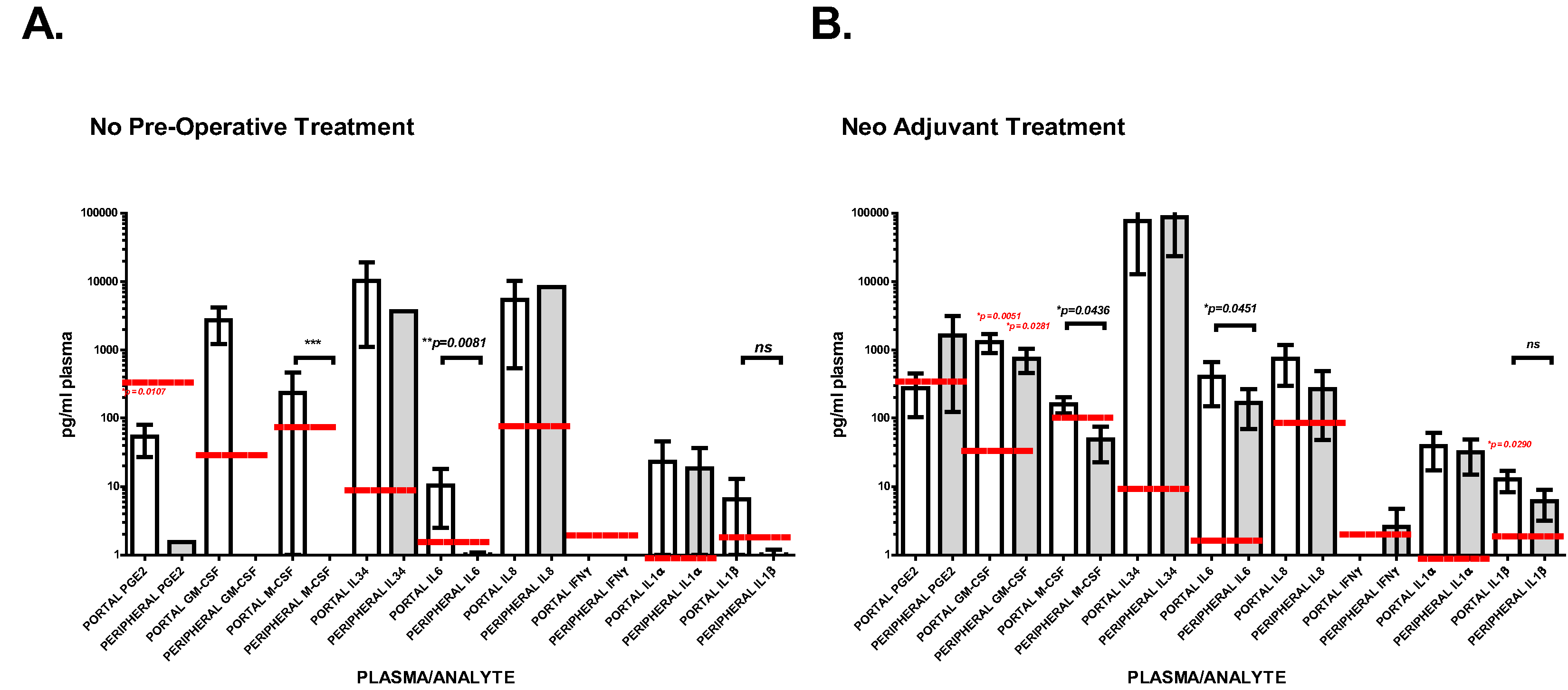

Supplement: S4 Fig — Data depicted are derived from Fig 2 Panel C. ELISA (Boster Scientific) analysis of matched portal (white bars) and peripheral (grey bars) plasma of 19 patients with adequate volume for extended analysis, with 16 who received neo adjuvant therapy (chemotherapy ± radiation therapy) prior to resection were compared for concentrations of PGE2, GM-CSF, M-CSF, IL-34, IL-6, IL-8, IFNγ, IL1α, and IL1β. (A) The three patients in this data set that did not receive neoadjuvant had high levels of M-CSF, IL-6, and IL1β in their portal blood plasma but little or no detected in their peripheral blood plasma. With so few untreated patient samples represented, no reliable statistical analysis was possible for comparison. For the 16 patients with neoadjuvant treatment (B), statistical differences were seen in increased M-CSF, IL-6, and IL1β concentrations in portal blood compared with peripheral blood plasma. Red lines indicate literature values for concentrations in non-malignant human plasma; p values in red indicate significant differences found in PDAC portal and peripheral blood plasma samples used in this study compared to control values cited in the literature. Sample bar indicates mean of values and error bars depict standard error of the mean, with * indicating p<0.05, ** p<0.01, ns = no significant difference seen, and *** indicate where samples with undetectable analyte levels precluded statistical comparison. (TIF) [file pone.0265725.s004.tif]
