# Supplementary material for: Pancreatic Ductal Adenocarcinoma (PDAC) circulating tumor cells influence myeloid cell differentiation to support their survival and immunoresistance in portal vein circulation
Source: PLoS One. 2022 Mar 22;17(3):e0265725. doi: 10.1371/journal.pone.0265725 (PMC8939813; doi:10.1371/journal.pone.0265725)
Supplement: S2 File — (HTML) [file pone.0265725.s006.html]

GENEWIZ NGS Differential Gene Expression Analysis Report


- Analysis Summary
- Differential Expression Analysis

# GENEWIZ NGS Differential Gene Expression Analysis Report

## 1. Analysis Summary

|  |  |
| --- | --- |
| Customer | Sally Litherland |
| Email | sally.litherland@adventhealth.com |
| Quote Number | 30-297546209 |
| Configuration | HiSeq 2x150 PE HO |
| Control | Oligo-9-060 |
| Treatment | Single-9-060 |

## 2. Differential Gene Expression Analysis

### 2.1 Experimental design

Samples listed in the table below were used in the following analysis.

### 2.2 Distribution of read counts

Distribution of read counts in libraries were examined before and after normalization. The original read counts were normalized to adjust for various factors such as variations of sequencing yield between samples. These normalized read counts were used to accurately determine differentially expressed genes. Figure 2.2.1 and Figure 2.2.2 show the read counts for each sample before and after the normalization.

Figure 2.2.1 Distribution of unnormalized reads count for genes

Figure 2.2.2 Distribution of normalized reads count for genes

### 2.3 Sample similarity assessment

Data quality assessments were performed to detect any samples that are not representative of their group, and thus, may affect the quality of the analysis.

The overall similarity among samples were assessed by the euclidean distance between samples. This method was used to examine which samples are similar/different to each other and if they fit to the expectation from the experiment design. The shorter the distance, the more closely related the samples are. Samples were then clustered by using the distance (Figure 2.3.1).

Figure 2.3.1 Heatmap of sample-to-sample distance

A principal component analysis is another way to reveal the similarity between samples based on the distance matrix. In Figure 2.3.2, samples were projected to a 2D plane spanned by their first two principal components. This type of plot is useful for visualizing the overall effect of experimental covariates and batch effects. The x-axis is the direction that explains the most variance and the y-axis is the second most. The percentage of the total variance per direction is shown in the label.

Figure 2.3.2 PCA analysis

### 2.4 Differentially expressed genes calling

Using DESeq2, a comparison of gene expression between the customer-defined groups of samples was performed. The Wald test was used to generate p-values and log2 fold changes. Genes with a p-value < 0.05 and absolute log2 fold change > 1 were called as differentially expressed genes.

The differentially expressed genes sorted by their p-value are shown in Table 2.4.2. If no differentially expressed genes were identified in the analysis, the top 500 genes with the most significant p-value are shown.

A bi-clustering heatmap was used to visualize the expression profile of the top 30 differentially expressed genes sorted by their p-value by plotting their log2 transformed expression values in samples (Figure 2.4.1). This analysis is useful to identify co-regulated genes across the treatment conditions.

Figure 2.4.1 Heatmap of top 30 differentially expressed genes

The global transcriptional change across the groups compared was visualized by a volcano plot (Figure 2.4.2). Each data point in the scatter plot represents a gene. The log2 fold change of each gene is represented on the x-axis and the log10 of its p-value is on the y-axis. Genes with a p-value less than 0.05 and a log2 fold change greater than 1 are indicated by red dots. These represent up-regulated genes. Genes with a p-value less than 0.05 and a log2 fold change less than -1 are indicated by green dots. These represent down-regulated genes.

Fig 2.4.2 Volcano plot

### 2.5 Gene ontology analysis

Significantly differentially expressed genes were clustered by their gene ontology and the enrichment of gene ontology terms was tested using Fisher exact test (GeneSCF v1.1-p2). Figure 2.5.1 shows gene ontology terms, if any, that are significantly enriched with an adjusted P-value less than 0.05 in the differentially expressed gene sets (up to 40 terms).

Fig 2.5.1 Gene Ontology Enrichment

- GENEWIZ Next Generation Sequencing
- Email: ngs@genewiz.com
- Phone: 908-222-0711 ext 1
