# Supplementary material for: Pancreatic Ductal Adenocarcinoma (PDAC) circulating tumor cells influence myeloid cell differentiation to support their survival and immunoresistance in portal vein circulation
Source: PLoS One. 2022 Mar 22;17(3):e0265725. doi: 10.1371/journal.pone.0265725 (PMC8939813; doi:10.1371/journal.pone.0265725)
Supplement: Attachment — Submitted filename: Pancreatic Ductal Adenocarcinoma (PDAC) Circulating Tumor Cells Influence Myeloid Cell Differentiation to Support their Survival and Immunoresistance in Portal Vein Circulation .pdf [file pone.0265725.s008.pdf]

Pancreatic Ductal Adenocarcinoma (PDAC) Circulating Tumor Cells Influence Myeloid Cell Differentiation to Support their Survival and Immunoresistance in Portal Vein Circulation

Summary of the research and overall impression

This work is focused on the study of the circulating tumor cells isolated from the portal blood. Most studies have focused on CTC detection and counting in peripheral blood samples but fewer reports have focused in capturing and detecting CTCs in vessels closer to the tumor, especially in the main veins that drain blood from the organ invaded by the cancer ('close-to-the-tumor liquid biopsy'). I consider this a very positive aspect of this approach. In this study portal vein CTCs were cultured ex vivo together with myeloids cells to study proliferation, motility and cluster formation compared to CTC alone. The interaction between these two cell types causes myeloid cells to differentiate into an immunosuppressive phenotype mediated by the release of M-CFS/IL-34 and IL-8.

Blocking this interaction would prevent this phenotype change in myeloid cells and is postulated as a possible treatment for pancreatic cancer, which is one of the most aggressive solid tumors for which there is little chance of success with current treatments.

The purpose of the research is important and of interest to potential readers, however, I have some doubts and problems in understanding the tests performed. My biggest doubt is related to the number of samples used in each assay, I don't quite understand the diversity in the samples used, especially in assays that do not depend on the existence of CTCs in the patient's blood. And also, the fact of assuming that BMC are M-FB, when in fact they are a heterogeneous mixture of different cell types.

The text is a bit difficult to follow as there are no clearly delimited sections, for example with a subtitle. The images in general could be improved in relation to the quality of the microscopy images and the size of the axes of the graphs which is sometimes difficult to see. Some images are poorly explained in the text.

Material and Methods:

CTC isolation method is based on the expression of epithelial cell markers, such as CK19 or EpCAM, couldn´t you have missed CTCs undergoing EMT?

Sample collection: The description of the patients indicates that 18 patients had received chemotherapy prior to surgery (Folfirinox or Gemcitabine/Abraxane) and 8 of them had even received radiation. It is described that the treatments will influence the release and the characteristics of CTC into the blood. This factor could be introducing noise in the results. I understand that the number of patients is low but it would be convenient for the authors to analyze the results separately for patients who have received therapy and those who have not. This may even give information on whether therapy affects the interaction of myeloid cells with CTCs.

I understand that patients will be in stage I or II disease if they are amenable to surgery but it might be useful to make this clear in the text. Inclusion criteria could be better described.

FACS: I have not checked all the antibody references but for example, for CD44 the references indicated correspond to CD34 and CD81 (line 110). Likewise, I don't know if the authors meant to refer to CD47 since CD44 is not among the markers used for cytometry of CTCs or M-FB.

RNA: Any assessment of RNA quality?

Results:

In Figure 1, I would suggest rearranging the panels in the order in which they are cited in the text. It is a bit odd to have to look at figure 1c first. This should be 1a if it is the first one being discussed.

Figures 1C-H how have they been taken? Why do we know they are CTC or M-FB? There are supposed to be two dyes but the image quality does not allow to see anything.

I find figure 1a rather incomprehensible. The authors claim that CTC-MFB co-culture induces CTC proliferation. I don't understand this result in the graph. I don't understand this result in the graph.

Of the 16 patients used in this trial, how many had received therapy? Does that affect the outcome?

Figure 2A is not referred in the text. I understand that for trials with CTCs the number of patients is not the total, but to analyze the levels of cytokines and growth factors in blood, why not all patients? What are the red stripes in figure 2A? Are they normal reference values? A bit unusual way to put them.

According to the text: "*GM-CSF levels in PDAC patient portal blood plasma were highly elevated compared to normal peripheral blood (p=0.0305) while M-CSF levels were moderately elevated over 247 normal (p=0.0383)"* Why do you compare portal vein levels with normal peripheral blood levels? You would have to compare portal blood GM-CSF levels with normal levels there and peripheral blood levels with normal levels there. Or are they the same?

Figure 2c would benefit from a legend stating that the white bars are portal blood and the gray bars are peripheral blood. Significant differences are marked between the two blood types but are they different from the reference levels in each compartment? Now, however, 24 patients are used for this figure. The criteria for this should be explained.

Here as before, treatments may have influenced the release of these factors into the blood.

Figure 3: The lettering on panels B-E is virtually unnoticeable.

One doubt that arises, perhaps because I have not understood it well, is that the authors refer to M-FB populations and blood mononuclear cells (BMC) indistinctly. But when isolated by FICOL BMC, this is a heterogeneous population of cells. In the test corresponding to Figure 3, they take BMC to study the interaction between CTC-MFB and in reality, it cannot be assumed that what is there is only M-FB.

Figure 3F is not clearly visible. Perhaps it could be divided into several graphs, one for each chemokine. Similarly, if all comparisons are made with the first graph, this can be indicated in the figure caption and there is no need to put so many dashes above the figure, you can put the asterisks indicating statistical significance only above each bar. This applies to other figures in the manuscript.

Figure 3M and 3N are vaguely explained in the figure legend. Annexin V plot should be supplemented.

Figure 4 is not explained in the text. It is very difficult to understand because it makes a statement and refers to the entire figure, panels A to D, without further explanation of what the different images show. What does zero mean, in the second bar of the graphs? For me, figure 4 does not contribute anything because I do not understand it.

Figure 5 is not explained either. This causes the reader's attention to be lost because it is very difficult to draw conclusions when they do not explain what they see in the graphs. It requires extra effort on the part of the reader.

Figure 6 focuses on gene expression changes in CTCs and M-FBs. In the materials and methods, the whole process of sequencing is described but in reality the only thing that is described is the expression of the same markers that were previously studied. The whole paragraph in materials and methods referring to the identification of differentially expressed genes does not make much sense with the results that are being presented in this article.

Figure 7A does not seem to have significant differences, but in the text the impression conveyed is that the treatment reduces the state of anergy ("treatment of PortalBMC cultures with anti-CSF1R, anti-IL-8, and/or anti-IL34 alone or in combination showed a 2-3-fold reduction of anergy among T cells). Perhaps this statement or the graph should be explained better.

Throughout the text there are a number of typos in the wording that could easily be corrected:

For example, in line 140 it says cells cells; 147 it says cluster clusters. Line 154 it does not have a verb (like were used)

Line 267, there is a dot after IL-8 that should be a colon.
